# Association of rituximab use with adverse events in adults with lymphoma or autoimmune disease: a single center experience

**DOI:** 10.3389/fmed.2025.1567886

**Published:** 2025-04-25

**Authors:** Mengsi Hu, Tingwei Zhang, Bing Liu, Qi Guo, Bing Zhao, Jiangong Lin, Zhimei Lv, Rong Wang

**Affiliations:** ^1^Department of Nephrology, Shandong Provincial Hospital Affiliated to Shandong First Medical University, Jinan, China; ^2^Department of Nephrology, Shandong Provincial Hospital, Cheeloo College of Medicine, Shandong University, Jinan, China; ^3^Department of Nephrology, The Affiliated Taian City Central Hospital of Qingdao University, Taian, China

**Keywords:** rituximab, adverse event, lymphoma, autoimmune disease, primary membranous nephropathy, adult, immunotherapy

## Abstract

**Objective:**

Rituximab (RTX) is a chimeric human/murine CD20 monoclonal antibody, which has been administered in treating hematological malignancies and various autoimmune disorders. This study aimed to present our center’s experience in RTX use in adults with lymphoma and autoimmune diseases (AID) including primary membranous nephropathy (pMN), as well as therapeutic effects of RTX on clinical outcome of pMN patients.

**Methods:**

A total of 761 Chinese patients were retrospectively included, who received RTX treatment at Shandong Provincial Hospital between January 1st, 2017 and December 31st, 2021, with person time of exposure spanning between their first dose of RTX and last follow-up date or the end of the study period.

**Results:**

Adverse events (AEs) occurred in 487 patients (64.0%), with a majority of infection (309, 40.6%) and a minority of non-infectious AEs (178, 23.4%); and the incidences of AEs were higher in lymphoma patients (381, 65.8%) than that in AID patients (106, 58.2%). Respiratory infections (215, 28.3%), gastrointestinal infections (49, 6.4%), urinary tract infections (41, 5.4%), cutaneous and mucosal infections (31, 4.1%), and infections in the abdominal cavity or pleurisy (4, 0.5%) were the leading types of infections. Cancer diagnosis [hazard ratio (HR), 3.926; 95% confidence interval (CI), 1.730–8.913] and prophylactic sulfamethoxazole/trimethoprim (SMZ/TMP) administration (HR, 3.793; 95% CI, 1.101–13.069) were associated with increased risk of infections. Immediate non-infectious AEs included anaphylaxis (44, 5.8%) and infusion reactions (99, 13.0%). Long-term non-infectious AEs included hypogammaglobulinemia (106, 28.6%), neutropenia (11, 5.5%) and interstitial lung disease (1, 0.1%). Female sex (HR, 0.515; 95% CI, 0.289–0.918) and cancer diagnosis (HR, 0.126; 95% CI, 0.049-0.323) were associated with higher risk of hypogammaglobulinemia. In 74 pMN patients, 13 (17.6%) patients experienced infections, with 2 cases of non-infectious AEs (2.7%). 6-month follow-up showed remission was achieved in 45 patients (60.8%), either as initial (61.0%) or alternative therapy (60.7%), without significant impacts on kidney function (*p* > 0.05).

**Conclusion:**

Our findings indicated AEs were common during RTX treatment, particularly in lymphoma patients, most of which were moderate and mild, highlighting a whole-process monitoring, timely interference and caring. And RTX was a safe and effective therapeutic option for pMN either as initial or alternative therapy in adult Chinese patients.

## Introduction

Rituximab (RTX) is a chimeric human/murine IgG1 monoclonal antibody, which binds to CD20 antigen on B cell surface, and kills B cells via multiple mechanisms including complement-dependent cytotoxicity (CDC), antibody-dependent cellular cytotoxicity (ADCC), induction of apoptosis and sensitization to chemotherapy (1–4). Intravenous administration of RTX results in rapid and sustained deletion of circulating and tissue B cells (5), which, since its first approval two decades ago, has revolutionized the therapeutic strategy of B-cell malignancies, and has achieved considerable efficacy benefits in treating a variety of autoimmune diseases (AID) and other disorders of immune dysregulation, singly or in combination with other agents (6–10). In spite of good tolerance and safety profile in the literature (11), RTX use may also come with a series of deleterious adverse drug effects including infusion reactions, anaphylaxis and infections (8, 12, 13), as well as some rare but serious events such as serum sickness, progressive multifocal encephalopathy and prolonged neutropenia (2, 11, 14–17). Occasionally, these adverse events (AEs) are atypical, highly diverse in different population and possibly difficult to predict, and thus may cause quandaries in treatment.

Primary membranous nephropathy (pMN) is a unique glomerular disease that is the most common cause of idiopathic nephrotic syndrome (NS) in adults (18). In recent years, pMN has been recognized as an AID caused by auto-antibodies targeting podocyte antigens, which leads to activation of complement and damage to the glomerular basement membrane (GBM) (19) and includes antigens such as phospholipase-A2 receptor (PLA2R) and thrombospondin type-1 domain-containing 7A (THSD7A) (20–23). RTX has been established as a key agent of many protocols for the treatment of glomerular diseases and antibody-mediated transplant disorders (24). And a growing body of data has indicated RTX therapy in treating refractory pMN patients who failed to respond to conventional immunosuppressive agents such as cyclophosphamide or calcineurin inhibitors with considerable remission rate (25–28). And since the release of clinical practice guidelines of Kidney Disease: Improving Global Outcomes (KDIGO) (2020) on glomerular diseases, RTX has been recommended as a first-line therapeutic option for moderate and high-risk pMN patients (29). Data regarding AEs associated with RTX use, the safety profile and therapeutic effects of RTX in pMN patients in the clinical practice are still under-reported.

Here in the present study, we reported immediate and long-term adverse drug events related to the clinical application of RTX in Chinese adult patients with lymphoma and AID in our hospital, and analyzed the associated risk factors. And we also investigated the safety and efficacy of RTX in pMN patients, which might increase the clinicians’ awareness of relevant AEs and caring for patients following courses of RTX, provide evidence for RTX use in the clinical practice of glomerular diseases, and to arouse some research interests in this filed.

## Materials and methods

### Study design

A cohort of adult patients from 18 to 88 years old, were included in this retrospective study, who received RTX treatment at Shandong Provincial Hospital between January 1st, 2017 and December 31st, 2021, with person time of exposure spanning between their first dose of RTX and last follow-up date or the end of the study period. In this study, median study follow-up after RTX administration for 761 patients was 24 months (IQR 21–24 months). Exclusion criteria: patients receiving transplant of either hematopoietic stem cell or solid organ, or other lymphodepleting therapy such as alemtuzumab, and those with primary immunodeficiencies or severe infection prior to the study.

### Data collection

Patients’ data were reviewed and extracted from patients’ electronic medical records, which included demographic information, allergy history, microbiology results and laboratory findings. And these records including documents of RTX infusions were also manually reviewed to identify relevant events, among which, adverse drug events and infections were classified and graded following Common Terminology Criteria for Adverse Events (CTCAE) version 5.0 (30). And events graded 3 or higher which required intravenous medications, hospitalization or prolonged hospitalization were characterized as “severe” in the present study. Anaphylaxis was defined according to European Academy of Allergy and Clinical Immunology (EAACI) guidelines: Anaphylaxis (2021 update) (31).

For patients with pMN, their renal pathology, laboratory tests of serum albumin, creatinine and PLA2R levels, as well as 24 h urine total protein quantity (24hUTP) were reviewed and collected. Secondary causes including potential malignancies, infections and other autoimmune diseases were excluded in all pMN patients. These patients received a total dose of 2 g RTX, administered as 375 mg/m^2^ weekly for four consecutive weeks per treatment course. Renal function was categorized by eGFR (mL/min per 1.73 m^2^) with chronic kidney disease epidemiology collaboration (CKD-EPI) formula. Patient’s outcome was reported as remission, including complete remission (CR) and partial remission (PR), non-remission (NR), or relapse. CR was defined by proteinuria less than 0.5 g/d, stable or improved renal function, and serum albumin 30 g/L. PR was designated by proteinuria between 0.5 and 3 g/d, stable or improved renal function, and serum albumin levels more than 30 g/L. NR was referred as 20% renal function deterioration and/or persistence of NS. And relapse was defined as reoccurrence of NS after CR or PR (32).

### Statistical analysis

For statistical analysis, quantitative data were reported as mean ± SDs. Statistical significance was assessed using Student’s *t*-test. Cox proportional hazards regression model was used for univariate and multivariate regression analyses. The chi-square test was used for correlation analysis. Data were analyzed using SPSS 25.0 software. *p* < 0.05 were considered to be statistically significant.

## Results

### Population characteristics

A total of 761 Chinese patients were included in the present study (Table 1), with a median age of 58.5 years at first dose. Of all these patients, 369 were female (48.5%), 748 were Han nationality (98.3%) and 13 minority nationality (1.7%). In our hospital, the indications for the use of RTX included lymphoma (579, 76.1%), and autoimmune diseases (182, 23.9%) including NS (90, 11.8%), neuro-optic myelitis (27, 3.5%), myasthenia gravis (22, 2.9%), systemic vasculitis (9, 1.2%), chronic inflammatory demyelinating polyneuropathy (8, 1.1%), nephritic syndrome (5, 0.7%), systemic lupus erythematosus (5, 0.7%), pemphigus vulgaris (5, 0.7%), autoimmune encephalitis (9, 1.2%), and multiple sclerosis (2, 0.3%). The majority of patients received multiple courses of RTX (482, 63.3%), and there were 163 (21.4%) or 116 (15.2%) patients who received a single dose or a single multidose course of RTX with a median of 2 doses per course (IQR 2–3 doses, range 2–5 doses), respectively. Pre-infusion prophylaxis was applied all patients, with corticosteroids and promethazine or antihistamines in 759 (99.7%) and 2 (0.3%) patients, respectively. Drug combinations during the study period included corticosteroids (536, 70.4%), hydroxychloroquine (2, 0.3%), cyclosporine A (2, 0.3%), tacrolimus (7, 0.9%), and sulfamethoxazole (41, 5.4%).

**Table 1:** Clinical characteristics and medication indications for RTX.

Clinical characteristics	Patient number (%)
Demographic characteristics	
Female sex	369 (48.5)
Age at dose, median (range)	58.5 (19–88)
Han nationality	748 (98.3)
Minority nationalities	13 (1.7)
Indications for RTX	
Cancer	
Lymphoma	579 (76.1)
Autoimmune diseases	
Nephrotic syndrome	90 (11.8)
Neuro-optic myelitis	27 (3.5)
Myasthenia gravis	22 (2.9)
Systemic vasculitis	9 (1.2)
Autoimmune encephalitis	9 (1.2)
Chronic inflammatory demyelinating polyneuropathy	8 (1.1)
Pemphigus vulgaris	5 (0.7)
Nephritic syndrome	5 (0.7)
Systemic lupus erythematosus	5 (0.7)
Multiple sclerosis	2 (0.3)
Dosing schedule	
Single	116 (15.2)
Single multidose course	163 (21.4)
Doses per course, median (IQR, range)	2 (IQR 2–3 doses, range 2–5 doses)
Multiple courses	482 (63.3)
Pre-infusion prophylaxis	
Corticosteroids_+_ promethazine	759 (99.7)
Promethazine+ antihistamines	2 (0.3)
Concurrent medications	
Corticosteroids	536 (70.4)
Hydroxychloroquine	2 (0.3)
Cyclosporine A	2 (0.3)
Tacrolimus	7 (0.9)
SMZ/TMP	41 (5.4)

SMZ/TMP, sulfamethoxazole/trimethoprim.

### Immediate and long-term AEs

Adverse drug events occurred in 487 patients (64.0%), with a majority of infection (309, 40.6%) (Table 2) and a minority of non-infectious AEs (178, 23.4%) (Table 3). And the incidences of AEs were higher in lymphoma patients (381, 65.8%, *n* = 579) than that in AID patients (106, 58.2%, *n* = 182). Respiratory infections (215, 28.3%), gastrointestinal infections (49, 6.4%), urinary tract infections (41, 5.4%), cutaneous and mucosal infections (31, 4.1%), and infections in the abdominal cavity or pleurisy (4, 0.5%) were the leading types of infections observed in the present study. Severe infections or lethal infections were observed in 62 (8.1%) or 7 patients (0.9%), respectively. The majority of lethal infections commonly occurred within first month after the drug use (5, 71.4%, *n* = 7) and were all respiratory infections. And more than half of infections occurred within first month after the first course of RTX injection (170, 55.0%, *n* = 309), which were dominated by respiratory infections (121, 71.2%, *n* = 170), with a relatively high incidence of severe infections (41, 24.1%, *n* = 170) (Table 2). And our data showed 257 of 579 (44.4%) patients with cancer (lymphoma), and 52 of 182 (28.6%) patients with non-cancer diagnosis (AID) experienced the complication of infection. In these lymphoma patients, there were 57 (9.8%, *n* = 579) or 6 (1.0%, *n* = 579) cases of severe or lethal infections, respectively; and three quarters experienced respiratory infections (193, 75.1%, *n* = 257). In patients with AID, 5 (2.7%, *n* = 182) patients had severe infections, and 1 (0.5%, *n* = 182) patients experienced lethal infections. And respiratory infections were the most common infectious complications in either lymphoma (193, 75.1%, *n* = 257) or AID (22, 42.3%, *n* = 52) patients.

**Table 2:** Adverse drug events among patients receiving RTX (infection).

Adverse drug events	Patient number (%)	Ca Patient number	AID Patient number
**Infection**	309 (40.6)	257	52
Infected sites			
Respiratory	215 (28.3)	193	22
Gastrointestinal	49 (6.4)	35	14
Urinary tract	41 (5.4)	30	11
Cutaneous and mucosal	31 (4.1)	24	7
Abdominal cavity or pleurisy	4 (0.5)	4	0
Degree of infection			
Any severity	309 (40.6)	257	52
Severe	62 (8.1)	57	5
Lethal	7 (0.9)	6	1
**Within first month**	170 (55.0)	149	21
Respiratory	121 (71.2)	113	8
Severe infection	41 (24.1)	39	2
Lethal infection	5 (71.4)	5	0

Bold values indicate the nature and timing of infection-related adverse reactions.

**Table 3:** Adverse drug events among patients receiving RTX (no-infection).

Adverse drug events	Patient number (%)	Ca Patient number	AID Patient number
**Non-infectious adverse events**	178 (23.4)	138	40
**Immediate events**	143 (18.8)	116	27
**Anaphylaxis**	44 (5.8)	36	8
**occurs during the first dose of medication**		30	8
**Infusion reactions**	99 (13.0)	80	19
Signs/symptoms			
Cough, wheezing, or dyspnea	28 (3.7)	23	5
Rigors	36 (4.7)	33	3
Hives, rash, or generalized pruritus	16 (2.1)	12	4
Chest or throat tightness	14 (1.8)	12	2
Headache	16 (2.1)	16	0
Fever	18 (2.4)	17	1
Nausea, vomiting, or abdominal pain	12 (1.6)	8	4
Transient hypertension	11 (1.4)	11	0
Arrhythmia	9 (1.2)	4	5
occurs during the first dose of medication	83 (83.8)	13	18
Termination	15 (2.0)		
**CTCAE grade ≥3 infusion reaction**	0	0	0
**Long-term events**			
**Hypogammaglobulinemia** ^**c**^	106 (28.6)	53	53
**Neutropenia** ^**d**^	11 (5.5)	4	7
Severe neutropenia	2 (1.0)	1	1
**Interstitial lung disease**	1 (0.1)	0	1

^cn = 371; dn = 199. Bold values indicate the types of non-infection-related adverse reactions.^

Cancer diagnosis (lymphoma) (HR, 3.926; 95% CI, 1.730–8.913; *p* = 0.001) and sulfamethoxazole/trimethoprim (SMZ/TMP) use for prophylaxis for pneumocystis jirovecii pneumonia (PJP) (HR, 3.793; 95% CI, 1.101–13.069; *p* < 0.05) were associated with increased risk of infections in the adjusted multivariable Cox proportional hazards models. In the present study, oral antimicrobial prophylaxis using SMZ/TMP was applied in patients with long-term administration of steroids and immunosuppressants (CD^4+^ < 200/mm or total lymphocyte count < 1,200/mm^3^) to prevent pneumocystis carnii pneumonia. Multivariate analysis that corticosteroid use was in association with increased risk of severe infections (HR, 2.705; 95% CI, 1.079–6.783; *p* < 0.05) (Table 4), and might be a significant factor in relation to infections (HR, 1.925; 95%CI, 0.998–3.713; *p* = 0.051) (Table 4).

**Table 4:** Cox proportional hazards models of risk factors for infection and severe infection.

Risk factor	Infection				Severe infection			
	Unadjusted model		Adjusted model		Unadjusted model		Adjusted model	
	HR (95% CI)	*p* value	HR (95% CI)	*p* value	HR (95% CI)	*p* value	HR (95% CI)	*p* value
Demographic characteristics								
Sex	1.085 (0.667–1.765)	0.742			1.359 (0.670–2.758)	0.395		
Nationality	2.044 (0.641–6.516)	0.227			0.048 (0.000–1,717.030)	0.570		
Age	1.036 (1.017–1.054)	<0.001	1.008 (0.988–1.027)	0.448	1.023 (0.999–1.049)	0.066	1.010 (0.983–1.038)	0.467
Diagnosis and rituximab dosing								
No. of doses	0.611 (0.467–0.798)	<0.001	0.995 (0.676–1.466)	0.981	0.514 (0.333–0.794)	0.003	0.623 (0.341–1.137)	0.123
Cumulative dose	0.999 (0.999–1.000)	0.002	0.999 (0.999–1.000)	0.148	0.999 (0.998–1.000)	0.014	1.000 (0.999–1.001)	0.398
Cancer diagnosis	6.939 (3.704–12.998)	<0.001	3.926 (1.730–8.913)	0.001	2.473 (1.198–5.106)	0.014	0.839 (0.306–2.301)	0.734
Concurrent medications								
Corticosteroids	4.073 (2.286–7.256)	<0.001	1.925 (0.998–3.713)	0.051	2.981 (1.371–6.480)	0.006	2.705 (1.079–6.783)	0.034
Tacrolimus	0.492 (0.068–3.550)	0.482			1.048 (0.143–7.685)	0.963		
SMZ/TMP	2.154 (0.676–6.865)	0.195	3.793 (1.101–13.069)	0.035	3.075 (0.733–12.899)	0.125	3.871 (0.852–17.584)	0.080
Cyclosporine A	0.049 (0.000–4,569.439)	0.606			0.049 (0.00–797,642.773)	0.722		
Intravenous immunoglobulin	0.777 (0.312–1.935)	0.588			0.642 (0.153–2.691)	0.545		

SMZ/TMP, sulfamethoxazole/trimethoprim.

More than three quarters of non-infectious AEs occurred in patients with lymphoma (138, 77.5%, *n* = 178), with rest in patients with AID (40, 22.5%, *n* = 178), most of which (143, 80.3%, *n* = 178) were captured during or immediately after the infusion of RTX, including anaphylaxis (44, 5.8%), and infusion reactions (CTCAE<3) (99, 13.0%) (Table 3). And most of anaphylaxis (36, 81.8%, *n* = 44), and infusion reactions (80, 80.8%, *n* = 99) were observed in patients with lymphoma. Anaphylaxis (38, 86.4%, *n* = 44) and infusion reactions (83, 83.8%, *n* = 99) mostly came along during the first dose, typically manifested as respiratory symptoms like cough or respiratory distress (18, 21.6%, *n* = 83), chills (33, 39.7%, *n* = 83) and skin involvement (16, 19.3%, *n* = 83). And there were infusion reactions of arrhythmia (5, 5.1%, *n* = 99) in AID patients. During the study period, there were anaphylaxis occurring in patients with lymphoma at as late as the third dose (2, 4.5%, *n* = 44) and infusion reactions at fourth dose (2, 2.0%, *n* = 99). For AID patients, there was only one case of infusion reactions after the first dose (at the second dose) (1, 1.0%, *n* = 99). Most patients who experienced these events (132, 92.3%, *n* = 143) were able to complete the dose after pausing the infusion, obtaining oxygen intake or giving antihistamines.

In the study period, long-term non-infectious AEs included hypogammaglobulinemia, neutropenia, and other adverse effect (interstitial lung disease in a patient with pMN). Rare events such as serum sickness or progressive multifocal encephalopathy was absent in this study. In 371 patients who were followed, hypogammaglobulinemia (<5 g/L) occurred in 76 patients (20.5%), of which more than half were with lymphoma (49, 64.5%, *n* = 76). And nearly half of these patients experienced hypogammaglobulinemia within 6 months (37, 48.7%%, *n* = 76). Of note, there was a notable proportion of pre-existing hypogammaglobulinemia in the follow-up population (52, 14.0%), of which more than half were with lymphoma (34, 65.4%, *n* = 52). 11 (5.5%) of 199 patients developed neutropenia, with two cases of severe neutropenia (<0.5×10^9^/L) (Table 3). Six out of 11 patients (54.5%) developed neutropenia during the first month post RTX administration, including four lymphoma patients and two AID patients, with a median onset of 1 month (IQR 1–4, range 1–5). Further, we analyzed risk factors of hypogammaglobulinemia in the regression analysis. It was shown that female sex and diagnosis of cancer (lymphoma) were in association with higher risks of hypogammaglobulinemia compared with males (HR, 0.515; 95% CI, 0.289–0.918; *p* < 0.05) and AID diseases (HR, 0.126; 95% CI, 0.049–0.323; *p* < 0.001), respectively (Table 5).

**Table 5:** Cox proportional hazards models of risk factors for hypogammaglobulinemia.

Hypogammaglobulinemia	Unadjusted model		Adjusted model	
Risk factor	HR (95% CI)	*p* value	HR (95% CI)	*p* value
Demographic characteristics				
Sex	0.576 (0.337–0.985)	0.044	0.515 (0.289–0.918)	0.024
National	1.465 (0.200–10.701)	0.707		
Year of age	0.998 (0.982–1.014)	0.797		
Diagnosis and RTX dosing				
No. of doses	1.446 (1.181–1.771)	<0.001	0.944 (0.706–1.262)	0.696
Cumulative dose	1.000 (1.000–1.001)	0.001	1.000 (1.000–1.001)	0.404
Cancer diagnosis	0.165 (0.074–0.367)	<0.001	0.126 (0.049–0.323)	<0.001
Concurrent medications				
Corticosteroids	0.870 (0.516–1.466)	0.601	1.419 (0.769–2.618)	0.263
Tacrolimus	1.492 (0.362–6.149)	0.580		
SMZ/TMP	2.415 (0.587–9.929)	0.222	0.991 (0.231–4.257)	0.990
Cyclosporine A	1.694 (0.234–1.694)	0.602		
Intravenous immunoglobulin	0.778 (0.311–1.950)	0.593		

Since the release of KDIGO clinical practice guidelines (2020) on glomerular diseases, RTX has been recommended as a first-line therapeutic option for moderate and high-risk pMN patients (29), whereas the profile of safety and therapeutic effects associated with RTX use in pMN patients was still largely based on previous clinical trials on RTX and clinical experience with other indications. In this study, we were interested to investigate the AEs associated with RTX use in patients with pMN, as well as the outcome of these patients in the clinical practice in our center. In the present study, 90 patients with NS were included. Of these patients, 74 (82.2%) patients, 55 (74.3%) males and 19 (25.7%) females, were pathologically diagnosed as pMN by renal biopsy, with elevated serum PLA2R levels in 61 patients (82.4%). All 74 pMN patients received a total of 2 g of RTX treatment.

Firstly, we found by chi-square test that there was a correlation between AEs in lymphoma patients and AEs in pMN patients following RTX use (*p* < 0.01). Similar to patients with lymphoma, the leading AEs in pMN patients were infections, which occurred in 13 (17.6%) patients and mostly occurred within first month after RTX use (10, 76.9%, *n* = 13), with four respiratory infections, three cutaneous infections and three other infections. Severe infections occurred in four pMN patients (5.4%), including respiratory (2, 50%, *n* = 4) and cutaneous infections (2, 50%, *n* = 4). Non-infectious AEs were noted in two pMN patients (2.7%). One suffered from chill, chest distress and pruritus during the first dose of RTX and completed the dose after giving antihistamines, and the other patients developed non-symptomatic interstitial lung disease, which was diagnosed by computed tomography (CT) and alleviated after treatment of corticosteroid (Table 6).

**Table 6:** Adverse drug events among pMN patients.

Parameters	Patients, No. (%)
Male sex	55 (74.3)
Female sex	19 (25.7)
Elevated serum PLA2R levels	61 (82.4)
**Infections**	13 (17.6)
**Within first month**	10 (76.9)
Respiratory	4 (40.0)
Cutaneous	3 (30.0)
**Severe infections**	4 (5.4)
Respiratory	2 (50.0)
Cutaneous	2 (50.0)
**Non-infectious AEs**	2 (2.7)
Chill, chest distress and pruritus	1 (1.4)
Interstitial lung disease	1 (1.4)

Bold values indicate the nature and timing of diverse reactions.

Next, we analyzed the clinical outcome of these pMN patients. Due to the relatively short period of RTX administration according to the KDIGO guideline in China and in our center, 6-month follow-up data were presented in this study. Our data showed that in these 74 pMN patients, clinical remission (CR + PR) was achieved in 45 patients (60.8%) and relapse was absent in the present study. Among which, 18 patients (24.3%) received initial RTX treatment, who previously received conservative therapy of angio-tension receptor blockers (ARBs). In this group, clinical remission was obtained in 11 patients (61.1%). Another group of alternative therapy of RTX included 56 pMN patients (75.7%), who previously received corticosteroids or in combination of immunosuppressants. Clinical remission was achieved in 34 patients (60.7%), comparable to initial therapy (Table 7). And in both groups, the follow-up data showed that the levels of 24UTP (*p* < 0.01) and serum PLA2R (*p* < 0.05) were remarkably reduced, with serum albumin levels significantly increased (*p* < 0.05) and eGFR unchanged (*p* > 0.05) (Table 8).

**Table 7:** Clinical outcome of patients with pMN at 6-month follow-up.

	Patient number (%)	CR	PR	NR
Initial therapy	18 (24.3)	4 (22.2)	7 (38.8)	7 (38.8)
Alternative therapy	56 (75.7)	15 (26.8)	19 (33.9)	22 (39.3)
Total	74	19 (25.7)	26 (35.1)	29 (39.2)

CR, complete remission; PR, partial remission NR, non-remission.

**Table 8:** Laboratory parameters in pMN patients at 6-month follow-up.

	Baseline	6-month follow-up	*p* value
Initial therapy			
Serum ALB (g/L)	23.65 ± 4.24	32.04 ± 6.86	0
eGFR	97.27 ± 21.10	101.13 ± 18.20	0.146
24UTP (g/24 h)	7.27 ± 4.14	3.58 ± 3.60	0.005
Serum PLA2R (Ru/mL)	129.00 ± 242.67	7.31 ± 14.73	0.038
Alternative therapy			
Serum ALB (g/L)	24.76 ± 6.27	34.85 ± 32.72	0.028
eGFR	76.15 ± 33.78	76.48 ± 32.49	0.883
24UTP (g/24 h)	7.18 ± 4.58	4.36 ± 5.38	0
Serum PLA2R (Ru/mL)	109.57 ± 193.90	17.13 ± 32.16	0.001

## Discussion

The present study reported immediate and long-term adverse drug events related to the use of RTX in an adult Chinese cohort in our hospital. Our findings showed that AEs occurred in more than half of the patients, including infections and non-infectious events, although pre-infusion prophylaxis was widely applied, and the incidences of AEs were higher in lymphoma patients than that in AID patients, suggesting AEs were common in patients receiving RTX treatment, particularly in lymphoma patients. The prevalence of infection was highly variable in different studies, ranging from 7.6 to 69.6% (7, 11, 33–35). This variation might be owing to the heterogeneity of the populations, treatment indications, and dosing regimens, as well as the study designs and implementation. Our data showed that infections were noted in 40.6% of these patients, with a small proportion of either severe infections (8.1%) or lethal infections (2.3%), lower than previously reported, where serious infection rated from 17.2 to 21.7% (36). The leading infectious complications following RTX use in the whole cohort and in the sub-cohort of either lymphoma or AID patients were respiratory infections, which was in accordance with prior studies (33, 37–39). In addition, we showed that the majority of infections, respiratory infections and lethal infections occurred during the first month after the administration, which were consistent with other data that most infections were found during the first 12 months (40, 41), emphasizing early and intense attention should be paid to during this period.

Since infections were the leading complication observed in the present study, we next investigated the associated risk factors. It was indicated that infection was independently associated with cancer diagnosis (lymphoma in this study) and prophylactic SMZ/TMP administration. In the literature, the infection risk of RTX therapy in patients with malignancy seemed to be quite controversial. Prior meta-analysis indicated adding RTX to chemotherapy for the treatment of hematological malignancies such as lymphoma would not increase any infection risk (42). In contrast, another study showed that non-Hodgkin lymphoma patients who was administered with long-term RTX treatment might have particular infection risk (43). Similar to the latter, our data supported an increased risk of RTX therapy in these patients with malignancy. In other words, patients with lymphoma might be particularly vulnerable to infection, compared to patients with other indications of AID. Accordingly, infection occurred in 44.4% of patients with lymphoma in this cohort, the proportion of which was greater than that of AID patients; and the incidence of severe or lethal infections in lymphoma patients were also higher. SMZ/TMP was for the primary prophylaxis for PJP in the non-malignancy population in this study with long-term steroids and immunosuppressants administration, and our results suggested that the benefit of SMZ/TMP prophylaxis in patients with RTX therapy might not outweigh the potential infection risks. In contrast, a recent retrospective study where SMZ/TMP prophylaxis might be correlated with reduced infection in patients receiving RTX treatment (44). Of note, in this study SMZ/TMP was for the primary prophylaxis for PJP in the non-malignancy population with long-term steroids and immunosuppressants administration, whereas this population per se was particularly susceptible to high risk of infections. From this point of view, it was not very clear that whether this high risk was due to the underlying diseases with related drug use or the SMZ/TMP administration, the correlation of which needed to be confirmed in larger scale, multi-center studies.

In this study, more than three quarters of non-infectious AEs occurred in patients with lymphoma. And infusion reactions and anaphylaxis were the leading immediate non-infectious AEs and mostly occurred in lymphoma patients, the incidence of which were particularly high during the first dose (both > 80%), and were also noted as late as the fourth dose, highlighting a consistent monitoring for these patients during the whole course. These results were in accordance with previous studies that these hypersensitivity reactions were more frequently observed in B-cell malignancies than in those with AID (45–47). These hypersensitivity reactions might be ascribed to a relatively higher proportion of cytokine release syndrome in patients with hematological malignancies (48, 49); and lower incidences of theses reactions might be correlated with prior long-term and concomitant use of corticosteroids in most AID patients (46). Despite this, most of these AEs, whether in lymphoma or AID patients, were moderate and mild, since most patients (92.3%) were able to complete the dose after timely intervention, without drug withdrawal; and lethal infusion reactions or anaphylaxis were absent in this study, implicating a relatively good safety profile of the drug for adult Chinese patients.

Hypogammaglobulinemia was one of the most common long-term non-infectious adverse sequelae from the RTX use (50, 51). There were studies showing that 30% of child patients experienced hypogammaglobulinemia following RTX treatment (52), In adults, the incidence seemed to be variable among different indications, from 10.3 to 56.0% in patients with malignancy or non-malignancy diseases (53–55). Very recently, studies have shown that the incidence of hypogammaglobulinemia reached as high as 63.3% in AID patients treated with RTX; and a significant decline in IgG and IgM levels was observed as early as 3 months after RTX initiation (51). In this study, 20.5% of followed patients developed this complication, and more than half were with lymphoma. However, this figure might be underestimated as gamma globulin monitoring was not a routine assessment in the clinical practice in different departments, and for some patients, and this might be refused due to personal economic reasons. In contrast, as pre-existing hypogammaglobulinemia was commonly seen among patients with either malignancy or AID (54–56), and for patients with NS, due to protein leakage from the urine, hypogammaglobulinemia tended to persist even after RTX administration (57), the development of hypogammaglobulinemia in this population might not be necessarily attributed to RTX alone and thereby led to the overestimation of the figure. Interestingly, our data showed that females were more likely to develop hypogammaglobulinemia after RTX use, which was in accordance with other studies (58, 59). This observation might be attributed to higher serum RTX exposure in females compared to males (59, 60), potentially associated with sex-based differences in drug metabolism, such as body weight distribution or hormonal influences. Additionally, the higher prevalence of AID in females may lead to a greater demand for RTX use in this population. Nevertheless, to date, the detailed mechanisms were still unidentified. Studies illustrated that hypogammaglobulinemia was more frequently observed in patients who received RTX compared with those who received chemoimmunotherapy or immunotherapy (54). Similarly, in this study, cancer diagnosis was also associated with increased risk of hypogammaglobulinemia in the whole cohort, which might be ascribed to high prevalence of pre-existing hypogammaglobulinemia and the potential perturbation of the immune system in patients with cancer (54, 56, 61).

RTX has progressively become a first line therapy for pMN in recent years. In our center, RTX therapy has been initiated for the treatment of NS from January 2021 since the release of KDIGO guidelines of glomerular diseases (2020). Our data showed a correlation between AEs in lymphoma patients and AEs in pMN patients following RTX administration. In consistent with the whole cohort and previous studies (62), in the sub-cohort of pMN, the leading AEs were infections, with a relatively lower incidence rate of 17.6% than the whole cohort. The major infectious complications of the sub-cohort were also respiratory infections, which was in accordance with prior studies (33, 37). Of note, non-infectious AEs were rarely observed in pMN patients; and long-term AEs such as hypogammaglobulinemia or neutropenia, and rare or lethal AEs were absent in this study. Slow infusion rates (6–8 h) in our center, detailed doctor-patient communication before the initiation of RTX therapy, and whole process monitoring and caring for patients during RTX administration might be of some help in identifying atypical symptoms, providing early interventions and lowering the incidences. In addition, in this study, RTX administration did not significantly affect the levels of eGFR. These results indicated a relatively good safety profile of RTX in the population of pMN, which, however, needed further confirmation through longer follow-up data.

Further, we reported the efficacy of RTX in pMN patients in our center at 6-month follow-up. Although relatively short, evaluation at this time point was still important as it reflected early remission of the disease and might alleviate the anxiety of patients with strong expectations of early therapeutic effects within several months. Our data showed that 60.8% of pMN patients achieved clinical remission, which was higher than a previous figure of 53% in a smaller sample-sized French cohort (63). And the remission rate of RTX use in pMN as initial therapy (61.1%) was comparable to alternative therapy (60.7%) at 6 months. This seemed to be different from previous data (37, 64), where a much higher remission rate of 73.1% for RTX as initial therapy at 12 months was reported (37). This inconsistency might be correlated with RTX pharmacokinetics in NS, as RTX bound to albumin in the blood, which could be eliminated by proteinuria, leading to decreased residual levels of the drug (63, 65–67). With the alleviation of the disease and recovery of the albumin, RTX levels might be affected, leading to varied remission at different disease stage. From this point of view, late remission cases and the remission rates were expected to increase through longer follow-up. Other potential mechanisms in association with resistance or failure of administering this drug have been proposed (68). In a retrospective study of 44 PMN patients, 10 (23%) demonstrated anti-RTX antibodies at 6 months post treatment (69), which was sufficient to block the cytotoxicity of RTX, regardless of complement activity. There were studies showing that RTX might be internalized into the B cell lysosome for degradation via forming complex with FcγRIIb (70, 71), and this the phenomenon has been observed in patients with rheumatoid arthritis and systemic lupus erythematosus (SLE) (72, 73). And moreover, it has been found that the number of B cells in the lymph nodes was not completely depleted despite the complete removal of circulating B cells by RTX (74), all of which might be playing a critical role in RTX resistance.

Of interest, a diversity of novel molecular mechanisms of MN has been uncovered recently, such as signaling pathways of TRAF6-TAK1, which was involved in pathogenesis of pMN through its interaction with TAK1 and downstream GSDMD/Caspase-1 axis-dependent podocyte pyroptosis (75). In patients with MN, Sirt6 deficiency, Wnt1/β-catenin pathway activation and RAS overexpression have been observed (76), and blockade of Wnt/β-catenin/renin-angiotensin system (RAS) axis attenuated podocyte damage and proteinuria in MN by Moshen granule, a proprietary Chinese medicine (77). IL-6/STAT3 pathway activation was another pivotal player in the pathogenesis of MN and was prohibited in podocytes by Mahuang Fuzi and Shenzhuo Decoction (MFSD) to achieve its therapeutic effects (78). In addition, microbial dysbiosis such as such Lactobacillus have also been identified in pMN, which might alleviate gastrointestinal toxicity of RTX by regulating the proinflammatory T cells in animal models (79, 80). To date, it was still unclear and undetected whether dysregulation of these signaling pathways or microbial dysbiosis played a role in the AEs or resistance of RTX in pMN, and if combined use of these drugs was more beneficial for pMN patients based on these observations to avoid AEs or enhance the efficacy of RTX, since the optimal dose of RTX remains problematic, all of which warranted further investigations.

To conclude, the current study indicated adverse AEs were common in adult Chinese patients receiving RTX treatment, most of which, however, were moderate and mild, implicating a good safety profile. And whole-process monitoring, timely interference and caring were important. The study also presented AEs in different indications including lymphoma and AID, and showed that lymphoma patients were prone to infectious and non-infectious AEs, in comparison with AID patients. Moreover, RTX was an effective and safe therapeutic option for pMN either as initial or alternative therapy. However, there were some limitations in this study. Firstly, this was a retrospective cohort, with the intrinsic weaknesses of bias due to the possible incompleteness in the medical records and the data collection, such as uncaptured subclinical or atypical AEs. Secondly, although the whole cohort was not a small one, the sub-cohort of pMN patients was relatively small, leading to failed analysis of risk factors associated with RTX use, and longer follow-up data would be beneficial for further evaluation and analysis. Furthermore, longer follow-up period might also be of significance for the whole cohort. Alternatively, large-scale prospective investigations were in need to further identify inconspicuous AEs and the associated risk factors, and thus to benefit patients by personalized RTX management with better outcome.

## Data Availability

The original contributions presented in the study are included in the article/supplementary material, further inquiries can be directed to the corresponding authors.
